# Transgenic mice overexpressing human ALOX15 under the control of the aP2 promoter are partly protected in the complete Freund’s adjuvant-induced paw inflammation model

**DOI:** 10.1007/s00011-023-01770-8

**Published:** 2023-07-27

**Authors:** Dagmar Heydeck, Kumar R. Kakularam, Dominika Labuz, Halina Machelska, Nadine Rohwer, Karsten Weylandt, Hartmut Kuhn

**Affiliations:** 1 Department of Biochemistry, Charité - Universitätsmedizin Berlin, corporate member of Freie Universität Berlin and Humboldt Universität zu Berlin, Charitéplatz 1, 10117 Berlin, Germany; 2 Department of Experimental Anesthesiology, Charité ˗ Universitätsmedizin Berlin, corporate member of Freie Universität Berlin, Humboldt-Universität zu Berlin, Hindenburgdamm 30, 12203 Berlin, Germany; 3 Division of Hepatology, Gastroenterology, Oncology, Hematology, Palliative Care, Endocrinology and Diabetes, Medical Department B, Brandenburg Medical School, University Hospital Ruppin-Brandenburg, Fehrbelliner Straße 38, 16816 Neuruppin, Germany; 4 Faculty of Health Sciences, Joint Faculty of the Brandenburg University of Technology Cottbus-Senftenberg, Brandenburg Medical School and University of Potsdam, Karl-Liebknecht-Straße 24-25, 14476 Potsdam, Germany; 5 Department of Molecular Toxicology, German Institute of Human Nutrition Potsdam-Rehbruecke, Arthur-Scheunert-Allee 114-116, 14558 Nuthetal, Germany

**Keywords:** Eicosanoids, Lipoxygenase, Inflammation, Colitis, Paw edema, Resolvins

## Abstract

**Background, objectives and design:**

Arachidonic acid 15-lipoxygenase (ALOX15) has been implicated in the pathogenesis of inflammatory diseases but since pro- and anti-inflammatory roles have been suggested, the precise function of this enzyme is still a matter of discussion. To contribute to this discussion, we created transgenic mice, which express human ALOX15 under the control of the activating protein 2 promoter (aP2-*ALOX15* mice) and compared the sensitivity of these gain-of-function animals in two independent mouse inflammation models with Alox15-deficient mice (loss-of-function animals) and wildtype control animals.

**Materials and methods:**

Transgenic aP2-*ALOX15* mice were tested in comparison with Alox15 knockout mice (Alox15^−/−^) and corresponding wildtype control animals (C57BL/6J) in the complete Freund’s adjuvant induced hind-paw edema model and in the dextran sulfate sodium induced colitis (DSS-colitis) model. In the paw edema model, the degree of paw swelling and the sensitivity of the inflamed hind-paw for mechanic (von Frey test) and thermal (Hargreaves test) stimulation were quantified as clinical readout parameters. In the dextran sodium sulfate induced colitis model the loss of body weight, the colon lengths and the disease activity index were determined.

**Results:**

In the hind-paw edema model, systemic inactivation of the endogenous Alox15 gene intensified the inflammatory symptoms, whereas overexpression of human ALOX15 reduced the degree of hind-paw inflammation. These data suggest anti-inflammatory roles for endogenous and transgenic ALOX15 in this particular inflammation model. As mechanistic reason for the protective effect downregulation of the pro-inflammatory ALOX5 pathways was suggested. However, in the dextran sodium sulfate colitis model, in which systemic inactivation of the Alox15 gene protected female mice from DSS-induced colitis, transgenic overexpression of human ALOX15 did hardly impact the intensity of the inflammatory symptoms.

**Conclusion:**

The biological role of ALOX15 in the pathogenesis of inflammation is variable and depends on the kind of the animal inflammation model.

## Introduction

The arachidonic acid 15-lipoxygenase (ALOX15) [1–4] is one of the six human arachidonic acid lipoxygenase isoforms and for each of these isoforms separate genes exist in the human genome [5]. In the mouse genome an orthologous gene was found for all human *ALOX* genes and in addition a functional *Aloxe12* gene was detected [6]. Its human ortholog is a corrupted pseudogene [6].

ALOX isoforms are fatty acid oxygenating enzymes, which convert polyunsaturated fatty acids to their corresponding hydroperoxy derivatives [7, 8]. In vivo the hydroperoxy lipids are rapidly converted to the more stable hydroxy derivatives or are converted to more complex oxygenation products exhibiting pro- or anti-inflammatory properties. Leukotrienes [9, 10] are classical pro-inflammatory ALOX products which are biosynthesized via the ALOX5 pathway. In contrast, lipoxins [11], resolvins [12, 13], maresins [14] and protectins [15] collectively called specialized pro-resolving mediators (SPMs) exhibit anti-inflammatory properties and have been suggested to play important roles in inflammatory resolution [16]. Although the biological roles of SPMs have recently been challenged [17, 18], there is a large number of reports describing their anti-inflammatory properties in sub-micromolar concentrations [16, 19]. In vivo SPMs can be formed via different biosynthetic pathways and the combined catalytic activities of various ALOX-isoforms (ALOX5, ALOX15, ALOX15B, ALOX12) have been implicated [17, 20].

Among the various mammalian ALOX isoforms ALOX15 orthologs are somewhat peculiar because they exhibit a high membrane oxygenase activity [21, 22]**.** The enzyme has been implicated in cell differentiation [23–25] and in the pathogenesis of various diseases [3, 26, 27]**,** and many of these data were obtained in genetically modified mouse models. For loss-of-function experiments, Alox15^−/−^ mice [28] have frequently been used in mouse models of human diseases [29–31]. For gain-of-function experiments, a number of transgenic mouse lines have been generated, in which mouse or human ALOX15 were overexpressed under the control of different promoters. Moderate overexpression of a transgenic mouse Alox15 construct that involved the mouse Alox15 promoter induced the formation of atherosclerotic lesions even when the mice were kept on a standard chow diet [32]. Transgenic expression of mouse Alox15 under the control of the alpha-cardiac myosin heavy chain promoter induced heart failure and cardiomyopathy [33, 34]. Transgenic overexpression of human ALOX15 under the control of the preproendothelin promoter induced lesion formation in LDL-receptor deficient mice [35] but it also inhibited tumor growth and metastasis in two different cancer models [36]. When human ALOX15 was transgenically expressed under the control of the scavenger receptor A promoter [37] the animals were protected from aortic lipid deposition and an augmented biosynthesis of pro-resolving mediators has been discussed as possible reason [38]. Transgenic mice, in which the human ALOX15 was expressed under the control of the villin promoter [39] were protected from the development of azoxymethane-induced colonic tumors [40]. More recently, a transgenic mouse line was generated, in which expression of the human ALOX15 was controlled by the Cre-lox promoter [41]. When these mice and corresponding wildtype controls were tested in a peripheral neuropathy model, no significant functional differences were observed between the two genotypes [41]. In rabbits, overexpression of human ALOX15 under the control of the lysozyme promoter induced macrophage-specific expression of the transgene [42] and protected the animals from aortic lipid deposition when fed a lipid rich Western-type diet [43]**.**

To explore the putative role of ALOX15 in adipogenesis [44] and in the pathogenesis of metabolic diseases [31, 45, 46]**,** we recently generated a transgenic mouse line, in which expression of human ALOX15 was controlled by the aP2 promoter [47]. This regulatory element directs transgene expression to adipocytes but also to hematopoietic cells such as bone marrow cells and peritoneal macrophages. These mice (aP2-*ALOX15* mice) are viable, reproduce normally but show subtle gender-specific differences when their bodyweight kinetics were followed during adolescence and early adulthood [47].

Since ALOX15 has previously been implicated in the pathogenesis of inflammatory diseases [3], we tested the aP2-*ALOX15* mice in two independent inflammatory mouse models. We found that aP2-*ALOX15* mice developed less pronounced inflammatory symptoms than corresponding wildtype control animals in the complete Freund’s adjuvant (CFA)-induced hind-paw inflammation model. In contrast, systemic functional inactivation of the Alox15 gene (*Alox15*^−/−^) induced more severe inflammatory symptoms and taken together these data suggest an anti-inflammatory role of Alox15 in this animal inflammation model. On the other hand, we did not observe significant differences between aP2-*ALOX15* mice and wildtype control animals in the dextran-sodium sulfate (DSS)-induced colitis model.

## Materials and methods

### Chemicals

The chemicals used for the different experiments were obtained from the following sources: phosphate buffered saline without calcium and magnesium (PBS) from PAN Biotech (Aidenbach, Germany); EDTA (Merck KG, Darmstadt, Germany), arachidonic acid (AA) and authentic HPLC standards of HETE-isomers (15R/S-HETE, 12S/R-HETE, 8R/S-HETE, 5S-HETE) from Cayman Chem (distributed by Biomol GmbH, Hamburg, Germany); acetic acid from Carl Roth GmbH (Karlsruhe, Germany); sodium borohydride from Life Technologies, Inc (Eggenstein, Germany); restriction enzymes from ThermoFisher (Schwerte, Germany). Oligonucleotide synthesis was performed at BioTez Berlin Buch GmbH (Berlin, Germany). Nucleic acid sequencing was carried out at Eurofins MWG Operon (Ebersberg, Germany). HPLC grade methanol, acetonitrile, n-hexane, 2-propanol, ethanol and water were from Fisher Scientific (New Hampshire, United States), incomplete Freund’s adjuvant (Fisher Scientific, New Hampshire, United States), *M. butyricum* desiccated (Fisher Scientific, (New Hampshire, United States), RNAlater (Sigma, Deisenhofen, Germany).

### Animals

Transgenic mice expressing the human ALOX15 under the control of the aP2 promoter were created as described before [47] and homozygous allele carriers were used for the experiments. A colony of homozygous Alox15^−/−^ mice [28] which were back-crossed into a C57BL/6J background at least 8-times, was maintained as inbred line in our animal house. All individuals were genotyped before they entered the experiment and ex vivo activity assays with peritoneal lavage cells and adipose tissue indicated functional disruption of the endogenous *Alox15* gene (*Alox15*^*−/−*^ mice) and overexpression of the human ALOX15 as transgenes (aP2-*ALOX15* mice).

### CFA-induced inflammation model and plethysmographic measurement of the paw volume

14–16 weeks old male mice of the different genotypes were used. The number of animals required for the experiments (6–8 animals per genotype) were determined by biometric calculations using previous results obtained with wildtype mice in the CFA-induced paw edema model. After the adaption period the hind-paw volumes of all animals (day 0) were determined (original hind-paw volumes). Then a CFA suspension (50 µg M*. butyricum* dissolved in 20 µl of incomplete Freund’s adjuvant) was injected into the foot pad of the right hind-paw of the animals. 20 µl of 0.9% NaCl solution was injected into the contralateral (left) hind-paw. One group of animals was sacrificed after 2 days (Fig. 2), for the second group the paw volumes were measured daily until day 14 after injection (Fig. 3) using a plethysmometer (model 37140, Ugo Basile, Gemonio, Italy). Two consecutive measurements (water displacement in ml) were carried out for each hind-paw and the mean of these two values was used for further evaluation. At the different time points after CFA- and NaCl injection the hind-paw volumes were determined again and the fold-change values of the hind paw volumes (quotients of the hind-paw volumes at the different time points divided by the original volumes of the corresponding hind-paws) were quantified as readout parameter for paw swelling. After the animals were sacrificed, a biopsy of the food pads was taken using a 6.0 mm biopsy punch. This biopsy material involved skin and the adjacent subcutaneous tissue. The biopsy material was cut into two pieces. One half of the biopsy was shock frozen in liquid nitrogen the other half was kept in RNAlater for gene expression studies.

### von Frey test

Pain intensity towards mechanic stimulation was tested using the von Frey test as described in Ref. [48]. The animals were kept in the test cages daily (1–2 times for 15 min) for accommodation, starting 6 days prior to nociceptive testing; they were individually placed in clear Plexiglas chambers located on a stand with anodized mesh (Model 410; IITC Life Sciences, Woodland Hills, Los Angeles, CA, USA). The sensitivity to mechanical stimulation was assessed using calibrated von Frey filaments in the range of 0.054 mN (0.0056 g) to 42.85 mN (4.37 g). The filaments were applied until they bowed, for approximately 3 s, to the plantar surface of hind-paws. The up-down method was used to estimate 50% withdrawal thresholds. Testing was started using a 2.74 mN (0.28 g) filament. Depending on the reaction of the animal, the next weaker or stronger filament was used. The maximum number of applications was 6–9, and the cut-off was 42.85 mN (4.37 g) according to previous studies. At the beginning of the experiments (day 0, prior to administration of CFA or NaCl) the original paw withdrawal thresholds was determined. At different time points after CFA/NaCl injection the paw withdrawal thresholds were determined again and the fold-change of the paw withdrawal threshold (quotient of the paw withdrawal threshold measured at the different time points divided by the original paw withdrawal thresholds) were determined as suitable readout parameter for pain sensation.

### Hargreaves test

Pain intensity towards thermal stimulation was measured using the Hargreaves test as described in Ref. [48]. Mice were accommodated to the device by placing them individually in clear Plexiglas chambers positioned on a stand with a glass surface (Model 336; IITC Life Sciences, Woodland Hills, Los Angeles, CA, USA), for the duration described under paragraph 4.4. To examine the heat sensitivity, radiant heat was applied to the plantar surface of the hind-paws from underneath the glass floor with a high intensity projector lamp bulb and paw withdrawal latency was evaluated using an electronic timer. The withdrawal latency was determined in two consecutive measurements separated by at least 10 s and afterwards the mean was calculated. For baseline determination, the heat intensity was adjusted to obtain a withdrawal latency of about 10–12 s for the control paws, and the cut-off was set at 20 s to avoid tissue damage. At the beginning of the experiments (day 0, prior to administration of CFA or NaCl) the original paw withdrawal latencies were determined. At different time points after CFA/NaCl injection the paw withdrawal latencies were determined again and the fold-change of the paw withdrawal latencies (quotient of the paw withdrawal latency measured at the different time points divided by the original paw withdrawal latency) were determined as suitable readout parameter for pain sensation. Mechanical (von Frey test) and heat sensitivity (Hargreaves test) were evaluated in the same groups of mice with an interval of at least one hour between the tests.

### DSS induced colitis model

Female 15–18-week-old wildtype (*n* = 10) and aP2-*ALOX15* mice (*n* = 10) were divided randomly into homogeneous groups according to their weight and age. The number of animals required for the experiments (*n* = 3 per genotype for the 0 time point, *n* = 4 per genotype for the 6 day time point, *n* = 3 per genotype for the 9 day time point) were determined by biometric calculations using previous results obtained with wildtype mice in the DSS-induced colitis model. Control (no colitis) wildtype and aP2-*ALOX15* mice were sacrificed on day 0 (*n* = 3/group). For induction of colitis, mice received 2.5% (wildtype/vol) dextran sodium sulfate (DSS; molecular weight = 36.000–50.000; MP Biomedicals, Eschwege, Germany) in the drinking water ad libitum for 5 days. Then DSS water was replaced by normal drinking water. On the 6th (*n* = 4/group) and 9th day (*n* = 3/group) wildtype and aP2-*ALOX15* mice were anesthetized with isoflurane and sacrificed by cervical dislocation. The colon was prepared, inspected for macroscopic signs of inflammation and colon length was determined. To assess colitis activity, body weight, stool consistency, and the presence of occult or gross blood were determined.

### RNA extraction and qRT-PCR

10–15 mg (wet weight) of inflamed paw tissue was prepared 2 and 14 days after CFA-injection and stored in RNAlater solution (Sigma-Aldrich/Merck, Taufkirchen, Germany) at – 20 °C. After thawing the tissue was cut into small pieces using a scalpel and then homogenized in 400 µl LBP buffer (Nucleospin RNA plus kit, Macherey–Nagel, Düren, Germany) using a FastPrep24 homogenizer. Cell debris was spun down and from the homogenate supernatant total RNA was extracted following the instructions of the vendor of the Nucleospin RNA plus kit (Macherey–Nagel, Düren, Germany). Subsequently, 500 ng RNA were reversely transcribed using the Tetro Reverse Transcriptase kit (Meridian Bioscience, Memphis, TN, USA, distributed by BioCat GmbH, Heidelberg, Germany) and Oligo dT_18_ reagents as recommended by the vendor. qRT-PCR was performed as described before [48]. Briefly, intron-spanning amplification primer combinations for mouse Alox5 were synthesized (BioTez GmbH, Berlin, Germany) and external amplification standards were prepared. The following amplification primer combination was used for mouse Alox5: 5’-TCG AGT TCC CAT GTT ACC GCT-3’ and 3’-CTG TGG TCA CTG GGA GCT TCG-5’. Expression of mouse Alox5 was quantified using standard curves (known copy numbers of the external amplification standards) and was normalized to Gapdh expression. qRT-PCR was performed on a Rotor Gene 3000 device (Corbett Research, Mortlake, Australia). Amplification products were generated and the progress of the amplification process was followed using the SensiMix™ SYBR PCR Kit (Meridian Bioscience, Memphis, TN, USA, distributed by BioCat GmbH, Heidelberg, Germany).

### Preparation of peritoneal lavage cells

For preparation of peritoneal macrophages, 10 ml of PBS was injected into the peritoneal cavity of sacrificed mice. The belly was gently massaged for 2 min and the fluid was removed puncturing the peritoneal cavity. Usually about 8–9 ml of cell suspension was recovered. Cells were spun down for 15 min at 800 g and were washed twice with PBS. Finally, the cells were reconstituted in 0.5 ml of PBS and were used for ex vivo ALOX activity assays.

### RP-HPLC analysis of the ALOX products

To quantify the amounts of ALOX products formed during the incubation period of the ex vivo activity assays a Shimadzu instrument (LC20 AD) equipped with a diode array detector (SPD M20A) was used and the hydroxy fatty acids were separated on a Nucleodur C_18_ Gravity column (Macherey–Nagel, Düren, Germany; 250 × 4 mm, 5 μm particle size) which was coupled with a guard column (8 × 4 mm, 5 μm particle size). The analytes were eluted isocratically using a solvent system consisting of acetonitrile: water: acetic acid (70: 30: 0.1, by vol) with a flow rate of 1 ml/min at 25 °C. The absorbance at 235 nm (absorbance maximum of the conjugated dienes) was recorded and UV-spectra of the dominant peaks recorded during the chromatographic runs were evaluated.

### Statistical evaluation of the experimental raw data

Statistical calculations and figure design were performed using GraphPad prism version 8.00 for Windows (GraphPad Software, La Jolla, CA, USA, www.graphpad.com). For statistical evaluation of the experimental raw data the Student’s *t*-test, the Mann–Whitney *U*-test and two-way repeated measure ANOVA were used.

## Results

### Transgenic aP2-ALOX15 mice express the transgene in bone marrow cells

The aP2 promoter [47] directs expression of the ALOX15 transgene to adipocytes and hematopoietic cells (bone marrow, peritoneal lavage cells). In mouse peritoneal lavage cells endogenous Alox15 is expressed at very high levels but other AA 12-lipoxygenating Alox-isoforms, in particular ALOX12, are not expressed. Thus, these cells are particularly suited for ex vivo activity assays suggesting the intracellular activity of the transgene. Since the transgenic human ALOX15 is an AA 15-lipoxygenating enzyme whereas the endogenous mouse Alox15 is AA 12-lipoxygenating it is possible to differentiate between the catalytic activity of the endogenous mouse Alox15 and the transgenic human ALOX15 on the basis of the reaction products.

**Fig. 1:**
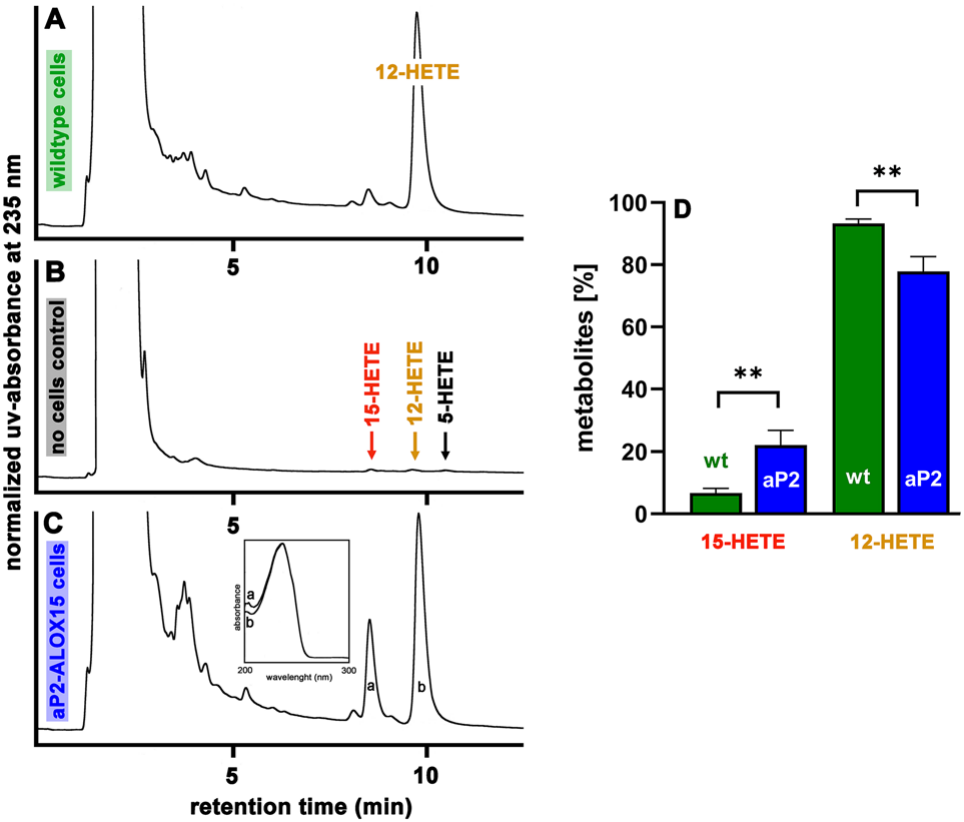
Ex vivo ALOX activity assays using peritoneal lavage cells of wildtype control mice (C57BL/6J) and aP2-*ALOX15* transgenic mice as enzyme source. Peritoneal lavage cells were prepared from three different mice (*n* = 3) of each genotype (C57BL/6J, vs. aP2-*ALOX15*) and ex vivo activity assays were carried out as described in the “Materials and methods” section. After lipid extraction the AA oxygenation products were analyzed by RP-HPLC. Representative chromatograms are shown. **A** Wildtype peritoneal lavage cells, **B** No cell control incubation, **C** aP2-*ALOX15* peritoneal lavage cells. Inset to panel **C** UV-spectra taken during chromatographic run of peaks a (15-HETE) and b (12-HETE), **D** Statistical evaluation of the chromatographic raw data. Metabolite composition formed by peritoneal lavage cells prepared from three wildtype C57BL/6J (wt) mice and three aP2-*ALOX15* transgenic animals was quantified. Means ± SD of the relative shares of 12- and 15-HETE were calculated for each metabolite. The degree of statistical significance **p < 0.01 was determined using unpaired *t*-test

When we incubated peritoneal lavage cells prepared from wildtype control mice with exogenous arachidonic acid, we found dominant formation of 12-HETE (Fig. 1A). Small amounts of 15-HETE were also detected and a similar product mixture has previously been reported for peritoneal lavage cells of wildtype mice [49]. When similar incubations were carried out in the absence of cells (Fig. 1B) or with a heat inactivated cell suspension (3 min at 90 °C) these products were not detected. Using peritoneal lavage cells of aP2-*ALOX15* mice for the ex vivo activity assays (Fig. 1C) 12-HETE was still the major reaction product but the relative share of 15-HETE formation was clearly increased. The chemical identity of the two major reaction products as 15-HETE (early eluting compound) and 12-HETE (late eluting compound) was confirmed by the UV-spectra taken during HPLC analysis using the diode array detector (insert to Fig. 1C). When we quantified the relatives shares of 12-HETE and 15-HETE formation by peritoneal lavage cells of aP2-mice and of wildtype controls (Fig. 1D) we found that the relative contribution of 15-HETE to the sum of the reaction products was 3.5-fold higher for peritoneal lavage cells prepared from aP2 mice (Fig. 1D). In contrast, the relative share of 12-HETE formation by aP2 cells was significantly reduced. The most plausible explanation for these differences is that the cells of the aP2 mice express in addition to the AA 12-lipoxygenating endogenous mouse Alox15 the AA 15-lipoxygenating transgenic human ALOX15 and previous qRT-PCR analyses have confirmed the expression of the transgenic ALOX15 in peritoneal lavage cells [47].

These data indicate the expression of the human ALOX15 transgene in peritoneal lavage cells. If one assumes that human ALOX15 and mouse Alox15 exhibit similar specific catalytic activities, the transgenic human ALOX15 should be expressed at about fivefold lower levels than the endogenous mouse Alox15 in peritoneal lavage cells. However, in other cells (bone marrow cells, adipocytes), in which expression of the endogenous mouse Alox15 is not as high as in peritoneal lavage cells, relative expression of the transgenic human ALOX15 is much higher than that of the endogenous mouse Alox15.

### Transgenic aP2-ALOX15 mice display diminished paw swelling in the CFA-induced paw inflammation model

**Fig. 2:**
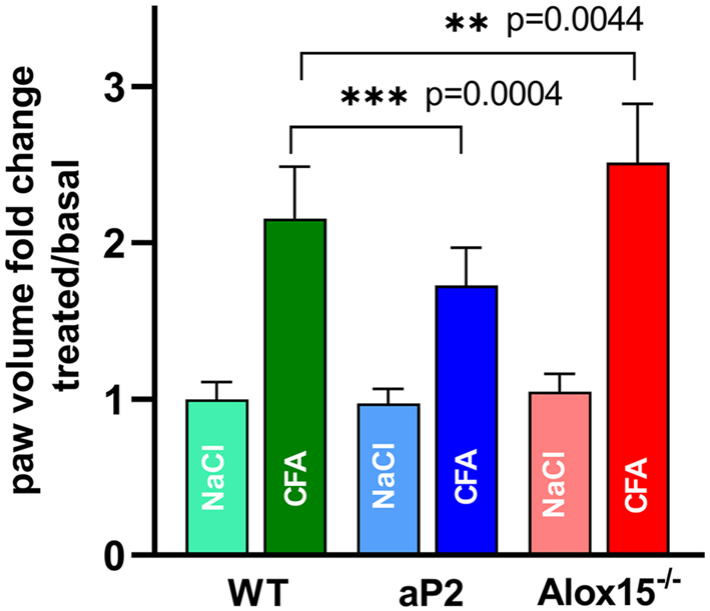
Transgenic expression of human ALOX15 lowered the intensity of CFA-induced paw swelling, but systemic inactivation of the endogenous *Alox15* gene induced an inverse effect. Genetically modified mice and corresponding wildtype control animals were maintained under standard conditions in the animal house of Charité. When entering the experiments, the volumes of their hind-paws were determined plethysmometrically (original hind-paw volumes). After these measurements a local inflammation was induced by injecting a suspension of CFA into the foot pad of the right hind-paw (see “Materials and methods” section). For control purposes a corresponding volume of isotonic NaCl solution was injected into the contralateral (left) hind-paw. After two days the volumes of the hind-paws were measured again and the degree of hind-paw swelling was quantified as hind-paw volume fold change (hind-paw volume two days after CFA or NaCl injection divided by the original hind-paw volume before CFA or NaCl injection). This fold-change was used as readout parameter to quantify the intensity of the inflammatory reaction. For each genotype 16–18 male individuals (15–16 weeks old) were examined in the frame of two independent experiments. The experimental raw data were combined and evaluated using the Mann–Whitney *U*-test. Means ± SD are shown and calculated *p* values are given

The CFA-induced paw inflammation model [50] is a sterile mouse inflammation model, in which an inflammatory reaction is induced in the hind-paw by injection of CFA. For control purposes an identical volume of isotonic NaCl solution is injected into the contralateral hind-paw of the same mouse. CFA injection triggers an inflammatory response that is characterized *inter alia* by an edema. This edema can be quantified by plethysmometric determination of the paw volume and the paw volume fold-change (quotient of paw volume after CFA or NaCl injection divided by the original volume of that paw before CFA or NaCl injection) can be quantified as suitable readout parameter for the intensity of the inflammatory reaction. As indicated in Fig. 2, CFA injection into the right hind-paw of C57BL/6J wildtype (WT) mice induced doubling of the paw volume two days after CFA injection. In aP2-*ALOX15* mice we also observed an increase of the paw volume, but here the degree of paw swelling was significantly less intense when compared with the wildtype mice (C57BL/6J). These data suggest that systemic expression of human ALOX15 in addition to expression of the endogenous mouse enzyme attenuated the intensity of the inflammatory response and thus, the transgenic enzyme may play an anti-inflammatory role in this mouse inflammation model.

If this conclusion is correct, systemic inactivation of the endogenous *Alox15* gene should induce more intense paw edema formation. To test this hypothesis, we examined Alox15^−/−^ mice and observed more pronounced edema formation in these mice when compared with C57BL/6J (wildtype) mice (Fig. 2).

**Fig. 3:**
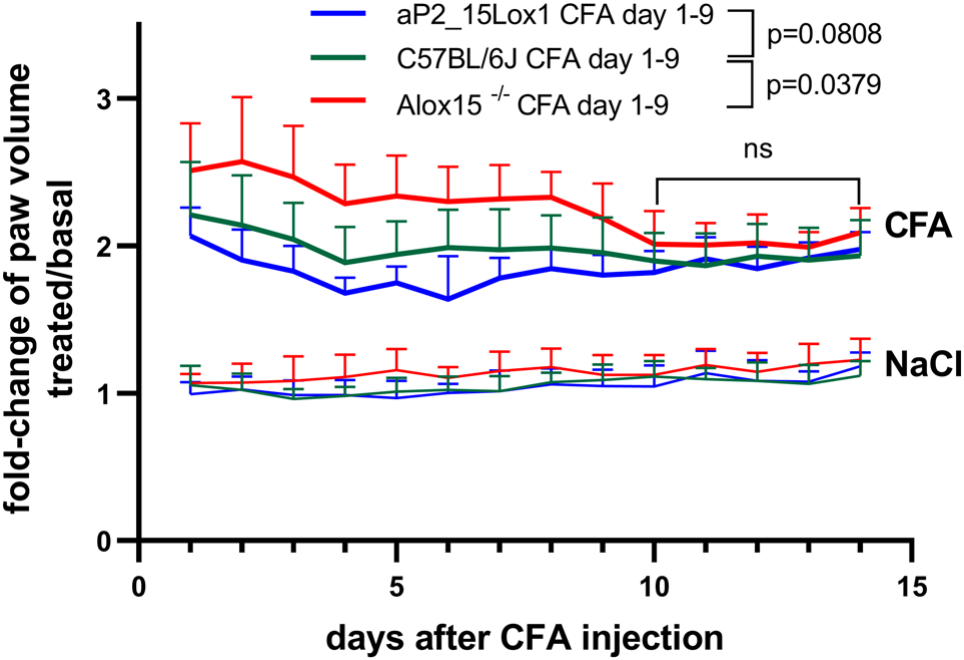
Transgenic expression of human ALOX15 reduced the intensity of CFA-induced paw swelling during the time-course of inflammation, but systemic inactivation of the endogenous Alox15 gene had opposite consequences**.** The CFA-induced hind-paw inflammation model was used (see “Materials and methods” section) and the volumes of the hind-paws (injected with NaCl or CFA) were determined each day plethysmometrically over the time period of 14 days. The degree of hind-paw swelling (quotient of hind-paw volume at the time points indicated after CFA or NaCl injection divided by the original hind-paw volume before CFA or NaCl injection, see Fig. 2) was quantified as readout parameter for the intensity of the inflammatory reaction. For each genotype 6–8 male individuals (14–16 weeks old) were included in this experiment. Means ± SD are given and the experimental raw data were statistically evaluated with two-way repeated measure ANOVA and *p* values are given

In an independent experiment we followed the kinetics of paw swelling during the time-course of the inflammation. From Fig. 3 it can be seen that the paw volume did not significantly change during the time-course of 14 days when NaCl (lower curves) was injected into the hind-paw of the animals of the three genotypes (C57BL/6J, aP2-*ALOX15*, Alox15^−/−^). In contrast, after injection of CFA into the hind-paw of wildtype mice (green trace in the upper curve triplet) we observed more than doubling of the paw volume one day after CFA injection (compared to the original paw volume before CFA injection) and this doubling is indicated by a fold-change of about 2. Interestingly, although the degree of paw swelling was reduced during the time-course of the experiment, it did not go back to the NaCl levels. This finding is consistent with the chronic character of the CFA-induced inflammation model, which will hardly resolve during the time-course of the experiment [51].

If one evaluates the kinetics of paw edema formation after CFA injection one can differentiate two different time windows. In the early time window (day 1–9) the three genotypes follow distinct edema kinetics. In fact, the blue curve representing the edema kinetics of the aP2-ALOX15 mice, was consistently below the green curve recorded for wildtype mice and statistical evaluation of the experimental raw data indicated a borderline significant difference (*p* = 0.0808, see Fig. 3). In contrast, the red curve that represents the edema kinetics for Alox15^−/−^ mice was consistently above the green curve (wildtype mice) and these data suggest a pro-inflammatory effect of Alox15 deficiency. The difference between these two genotypes (wildtype vs. Alox15^−/−^) was statistically significant (*p* = 0.0379). Interestingly, the differences between the three genotypes observed in the early time window (day 1–9) could not be confirmed at later time points of the experimental protocol. In fact, in the late time window (day 10–14) not significant differences in the extent of the paw edema was seen when the three genotypes were compared.

Taken together, these data suggest that transgenic expression of human ALOX15 under the control of the aP2 promoter attenuated the degree of inflammation in the CFA model. In contrast, systemic inactivation of the endogenous Alox15 gene rendered the mice more sensitive for CFA-induced paw swelling. These results are consistent with an anti-inflammatory activity of ALOX15 in this particular inflammation model. It should, however, been stressed that the phenotypic alterations were only observed during the early stages (1–9 days) of the inflammation process. At later stages (10–14 days) there were hardly any differences between the three genotypes.

### Transgenic aP2-ALOX15 mice display reduced mechanical sensitivity in the CFA-induced inflammation model (von Frey test)

Pain (*dolor*) is one of the cardinal symptoms of inflammation and inflamed tissue exhibits an elevated pain sensitivity. In other words, the degree of pain intensity may be considered a suitable readout parameter for the severity of inflammation. To test the pain sensitivity of the inflamed hind-paw following mechanical stimulation we carried out the von Frey test. As readout parameter, we determined the withdrawal threshold of the inflamed hind-paw in g. The lower the hind-paw withdrawal threshold, the higher the pain sensitivity of the inflamed paw.

**Fig. 4:**
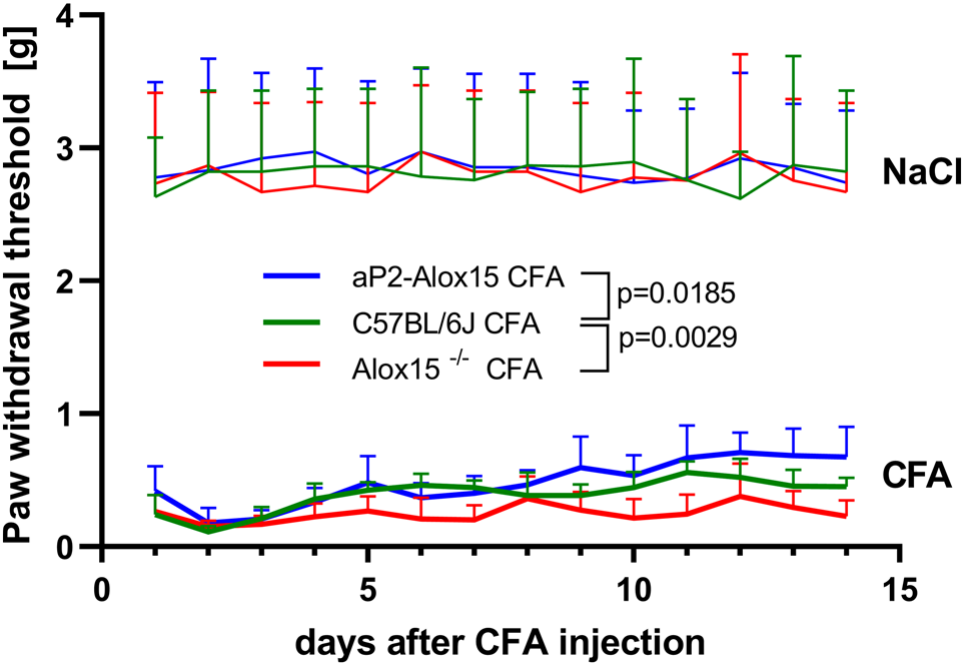
Transgenic expression of human ALOX15 reduced mechanical sensitivity in the CFA-induced inflammation model, but systemic inactivation of the endogenous Alox15 gene had opposite functional consequences. The CFA-induced hind-paw inflammation model was used (see “Materials and methods” section) to test the sensitivity of the inflamed hind-paw to mechanical stimulation (von Frey test, see “Materials and methods” section). The paw withdrawal threshold (in g) was determined for the hind-paws of all animals at the time points indicated. This procedure was repeated daily over the entire time frame of the experiment (day 1–14). For each genotype 6–8 male individuals (14–16 weeks old) were included. Statistical evaluation of the experimental raw data was carried out with two-way repeated measure ANOVA. Means ± SD at each time point and *p* values are given

From Fig. 4, it can be seen that the CFA treatment resulted in the increased sensitivity to von Frey mechanical stimulation of the inflamed paw and that the degree of the sensitivity was significantly lower for aP2-*ALOX1*5 mice when compared with wildtype controls. In fact, in average the blue curve is above the green curve at most time points of the experiments and statistical evaluation of the experimental raw data suggested a significant difference (*p* = 0.0185) between the two genotypes. In contrast, *Alox15*^*−/−*^ mice showed a higher mechanical sensitivity since the red curve is below the green curve at most time points of the experiment. Here again, statistical evaluation of the experimental raw suggested a significant difference (*p* = 0.0029). These data are consistent with the paw volume data, which are illustrated in Fig. 3.

On the other hand, we observed an interesting difference between the kinetics of the paw volume alteration (Fig. 3) and the sensitivity of the mice for mechanical stimulation of the pain sensation (Fig. 4). When the paw volume was used as readout parameteraP2-ALOX15 mice were only protected during the early phases of the experiment (day 1–9). At later stages (days 10–15) there was no differences any more when wildtype mice and aP2-ALOX15 mice were compared (Fig. 3). When the sensitivity of the animals for mechanical stimulation of pain sensation was used as readout parameter (Fig. 4) inversed kinetic were observed. Here aP2-ALOX15 mice were only protected at later time points (days 8–15) whereas no differences were detected at earlier stage (days 1–7) of the inflammation reaction. The molecular basis for the different kinetics of the two different readout parameters (extent of the inflammatory edema vs. inflammatory pain) has not been explored. However, it might be possible that in this particular inflammation model formation of the inflammatory edema, which is a direct consequence of fluid transfer from the blood stream into the inflamed tissue, is a fast process that rapidly follows CFA injection. In contrast, induction of inflammatory pain might be more time consuming and will take a couple of days to fully develop.

### Transgenic aP2-ALOX15 mice display reduced heat sensitivity in the CFA-induced inflammatory model (Hargreaves test)

To obtain additional measures for pain sensitivity of the inflamed paw we applied the Hargreaves test that quantifies the heat sensitivity of the inflamed tissue. For this purpose, a CFA suspension was injected into the right hind-paw pad and NaCl into the left hind-paw pad of the mice (day 0). At the time points indicated the hind-paw withdrawal latency was quantified as measure of pain sensitivity.

**Fig. 5:**
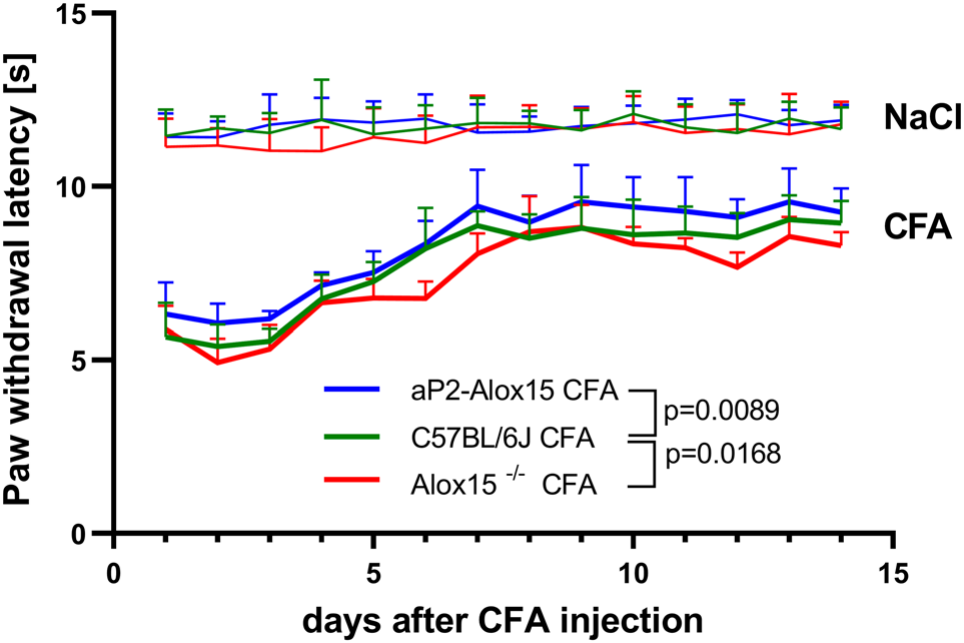
Transgenic expression of human ALOX15 reduced the sensitivity to heat in the CFA-induced inflammation model, but systemic inactivation of the endogenous Alox15 gene had no functional consequences. The CFA-induced hind-paw inflammation was induced as described in “Materials and methods” section and the sensitivity of the inflamed tissue to heat was analyzed using the Hargreaves test (see “Materials and methods” section). The paw withdrawal latencies were determined as readout parameter. For each genotype 6–8 male individuals (14–16 weeks old) were included in the experiment and individual paw withdrawal latencies were determined each day. Statistical evaluation of the experimental raw data was carried out with two-way repeated measure ANOVA. Means ± SD are shown at each time point and *p* values for comparison of the different genotypes are given

As indicated in Fig. 5 CFA treatment reduced the paw withdrawal latency for all genotypes (compared with NaCl injected hind-paws) and these data indicate that the CFA-injected hind-paws were more sensitive to heat than their NaCl injected counterparts. Most interestingly, the degree of sensitization was significantly lower for aP2-*ALOX15* mice when compared with wildtype animals. In fact, the blue curve in Fig. 5 (lower curve triplet), which represents the heat sensitivity of aP2-ALOX15-KI mice, was above the green curve (wildtype control mice) at most time points of the experiment and we observed a significant difference (*p* = 0.0089) between the two genotypes. In contrast, when we compared the paw withdrawal latencies of Alox15^−/−^ mice (red curve) with the corresponding data of wildtype control animals (green curve) we found that the red symbols were below the green symbols at most time points of the experimental protocol and statistic evaluation of the experimental raw data indicated a significant difference (*p* = 0.0168) between the two genotypes. These data confirm the observation made in the von Frey test (Fig. 4).

### Expression of the pro-inflammatory Alox5 is reduced in aP2-ALOX 15 transgenic mice

We have previously reported that systemic disruption of the mouse Alox15 gene activated the Alox5 pathway in peritoneal lavage cells and bone marrow cells [47]. In contrast, transgenic expression of human ALOX15 inactivated the Alox5 pathway in these two genotypes [47]. If this regulatory response does also occur in other pro-inflammatory cells such as neutrophils, monocytes or lymphocytes, the inactivation of the Alox5 pathway might contribute to the anti-inflammatory effect of transgenic expression of human ALOX15 in aP2-*ALOX15* mice.

**Fig. 6:**
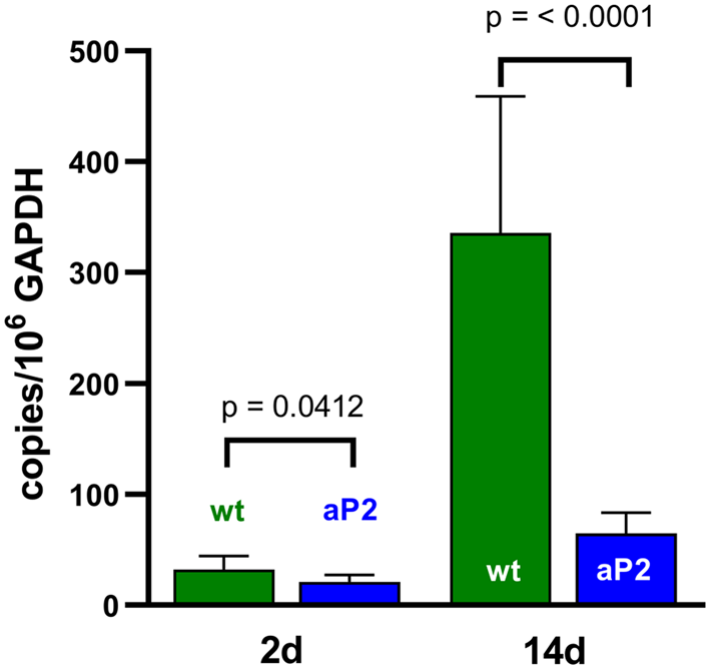
Expression of Alox5 mRNA in the inflamed paw tissue of wildtype mice and ap2-*ALOX15* mice in the CFA-induced inflammation model. The CFA inflammation was induced as described in “Materials and methods” section. Inflamed tissue was prepared from 5 animals of each genotype 2 and 14 days after CFA administration and stored in RNAlater at -80° C. After thawing total RNA was extracted, aliquots were reversely transcribed and qRT-PCR was carried out employing external amplification standards for Alox5 and Gapdh. The Alox5 mRNA copy numbers were quantified per 10^6^ Gapdh mRNA copy numbers and two independent PCR runs were carried out for each RNA extract. The experimental raw data were statistically evaluated with the Student’s *t*-test and the *p* values for the comparison of the two genotypes are given. Means ± SD are shown

To test this hypothesis, we quantified the expression of Alox5 mRNA in the inflamed paw tissue of wildtype mice and aP2-*ALOX15* mice at day 2 and day 14 following CFA administration. From Fig. 6 it can be seen that 2 days after CFA injection Alox5 mRNA was only present at low levels in the inflamed paw of wildtype mice. In contrast, much higher expression levels of Alox5 mRNA were detected at day 14 suggesting that in this particular inflammation model Alox5 does not belong to the immediate early genes of inflammation. Next, we compared the Alox5 mRNA expression levels in CFA-treated wildtype mice and aP2-*ALOX15* mice and found that 2 days after CFA injection Alox5 expression was higher in wildtype mice when compared with aP2-*ALOX15* animals and the difference was statistically significant. 14 days after CFA injection, Alox5 mRNA levels were more than sevenfold elevated in wildtype mice when compared with aP2-*ALOX15* (*p* < 0.0001) (Fig. 6).

In summary, one can conclude that transgenic expression of human ALOX15 in the CFA-induced inflammation reduces the expression levels of the pro-inflammatory Alox5 pathway and this regulatory response might contribute to the anti-inflammatory effect of transgenic ALOX15 expression observed in the CFA inflammation model (Figs. 2, 3, 4, and 5).

### Transgenic ALOX15 expression does hardly impact the inflammatory response in the DSS colitis model

DSS colitis is a frequently employed mouse model of gut inflammation [52, 53] and we have previously reported that systemic inactivation of the Alox15 gene protected female mice from the development of inflammatory symptoms [54]. However, when Alox15-deficiency was crossed into an *fat1* overexpressing background the protective effect of fat1 overexpression was reduced [55]. Thus, systemic inactivation of the endogenous Alox15 gene indicated antagonistic effects in wildtype and fat1 overexpressing mice.

**Fig. 7:**
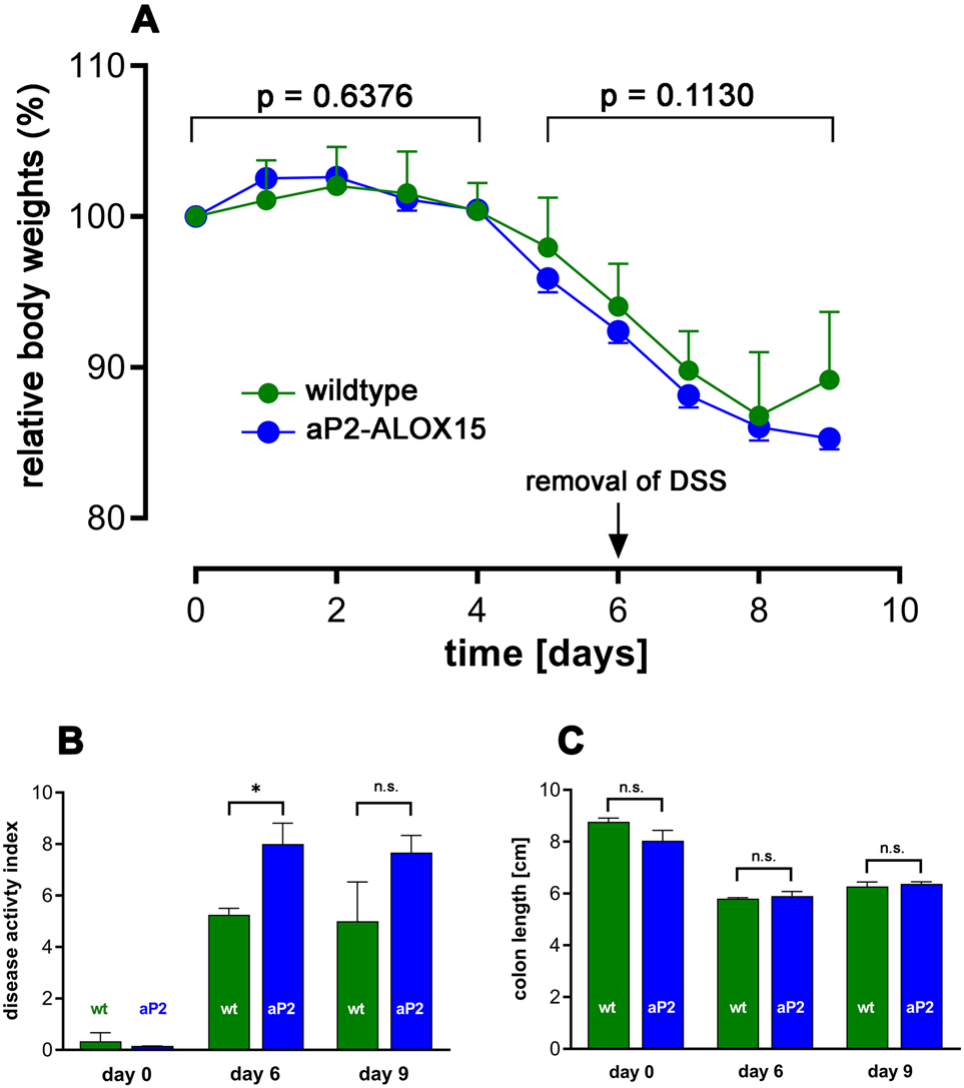
Transgenic expression of human ALOX15 under the control of the aP2 promoter did hardly impact the intensity of inflammatory symptoms in the DSS-colitis model. DSS-colitis was induced in 10 female wildtype (C57BL/6J, 15–18 weeks old) mice and 10 female aP2-*ALOX15* animals (same age) by daily administration of 2.5% DSS in the drinking water for five consecutive days. Before entering the experiments (first administration of DSS) the absolute body weights of all animals were determined. The means of these body weights for each genotype were set 100% and the relative individual body weights were determined each day. For control measurements three individuals of each genotype were sacrificed and the readout parameters (disease activity index, colon lengths) were quantified. For the remaining seven individuals of each genotype the relative body weight kinetics were determined and at day 6 of the experimental protocol the DSS containing drinking water was removed. Four individuals of each genotype were sacrificed, colon lengths and DAI were determined and the remaining three individuals of each genotype were kept on normal drinking water for additional 3 days. Finally, the remaining animals were also sacrificed and the DAI and colon lengths were quantified. Means ± SD are given and statistical evaluation was carried out using two-way repeated measure ANOVA. **A** Body weight kinetics, **B** Disease activity index, **C** colon length

To explore the impact of transgenic expression of human ALOX15 in the mouse DSS-colitis model we administered to the mice 2.5% DSS in the drinking water for five consecutive days. Afterwards the DSS-containing drinking water was replaced with fresh drinking water and the body weight kinetics of wildtype mice and aP2-*ALOX15* animals were recorded over a time period of 9 days. From Fig. 7A, it can be seen that both, wildtype mice and aP2-*ALOX15* mice, started losing body weight at day 5 of the experimental protocol and this loss of body weight is a consequence of enteral loss of body fluid. During this time period there was no difference between the two genotypes (*p* = 0.6376). Interestingly, at later time points the bodyweight curves for the two genotypes deviated from each other and aP2-*ALOX15* mice showed a more severe losses of body weight. In fact, the green bodyweight curve (wildtype mice) was consistently above the blue curve (aP2-*ALOX15* mice) and statistical evaluation (two-way repeated measure ANOVA) of the two curves suggested a borderline significance (*p* = 0.1130). According to these data aP2-*ALOX15* mice lose more body weight than the wildtype control animals and thus, transgenic expression of human ALOX15 intensifies the inflammatory symptoms. In other words, expression of the transgene induces a pro-inflammatory effect.

When the disease activity index (DAI) was measured as readout parameter for the intensity of enteral inflammation (Fig. 7B) we found that at day 6 and day 9 aP2-*ALOX15* mice showed increased DAI values compared to wildtype mice. The difference was statistically significant at day 6 but not significant at day 9 (*p* = 0.2500). No significant differences between the two genotypes were observed when the colon lengths were compared (Fig. 7C). Taken together, these data suggest that transgenic expression of human ALOX15 under the control of the aP2 promoter does not protect from DSS-induced colitis as it was the case in the FCA paw edema model (Figs. 2, 3, 4, and 5). In fact, in the DSS-colitis model (Fig. 7A–C) we rather observed a trend for a pro-inflammatory effect of aP2-*ALOX15* transgene expression.

## Discussion

### Degree of novelty and advancement of knowledge

Mammalian ALOX15 orthologs have been implicated in cell differentiation [23–25] and in the pathogenesis of inflammatory [3, 56], hyperproliferative [57, 58] metabolic [31, 59] and neurological diseases [60, 61] but the precise role of the enzyme in inflammation is far from clear. In different animal inflammation models pro- and anti-inflammatory roles have been suggested for Alox15 and most of these in vivo data originate from loss-of-function studies using Alox15^−/−^ mice [28] or gain-of-function research strategies employing different types of transgenic Alox15 overexpressing animals. To explore the biological role of ALOX15 in endothelial cells, expression of an ALOX15 transgene construct was regulated by the preproendothelin promoter [35, 36]. For functional studies investigating the biological role of the enzyme in macrophages the promotor sequences of the lysozyme [38] or the scavenger receptor A genes [37] were used. For cell specific expression of the ALOX15 transgene in gut epithelial cells the villin promoter was employed [39, 62]. However, cell-specific expression the an ALOX15 transgene in adipocytes has not been reported so far. We recently created transgenic mice which express human ALOX15 under the control of the aP2 promoter and these mice overexpress human ALOX15 in adipocytes of white adipose tissues [47]. Expression of endogenous Alox isoforms in adipose tissue is minimal and thus, our aP2-*ALOX15* mice are particularly suited to study the potential role of *ALOX15* in adipose tissue. When we profiled transgene expression in other cells of the aP2-*ALOX15* mice we observed high levels of ALOX15 mRNA and catalytic activity in hematopoietic cells such as bone marrow cells and macrophages [47]. Since macrophages play an important role in the pathogenesis of inflammatory diseases, we tested the aP2-*ALOX15* mice in two types of animal inflammation models and found that transgenic expression of human ALOX15 under the control of the aP2 promoter partly protected the mice from the development of inflammatory symptoms in the CFA-induced hind-paw inflammation model (Figs. 2, 3, 4, and 5) and these data suggest an anti-inflammatory role of the transgenic enzyme. However, in the DSS-induced colitis model transgene expression did not reduce the intensity of the inflammatory symptoms. In fact, we even observed a trend for more intense inflammatory symptoms in aP2-*ALOX15* mice, but the differences to wildtype control individuals were not statistically significant (Fig. 7A, B). If one puts the experimental data obtained with the aP2-*ALOX15* mice into the context of previous literature reports on the potential role of ALOX15 in inflammation one may conclude that the enzyme may exhibit pro- and anti-inflammatory properties depending on the type of the animal inflammation model.

### Limitations of the study

The aP2-*ALOX15* mice used in this study have been characterized comprehensively with respect to their basic biological characteristics [47]. They are viable, reproduce normally and do not show major developmental defects. Although gender-specific differences were observed when the body weight kinetics of aP2-*ALOX15* mice and wildtype control animals were recorded during adolescence and early adulthood. The differences were rather subtle and hardly impacted post-partal development [47]. However, the most serious limitation of these mice is their mixed genetic background. Although we determined the site of transgene incorporation into the genome and although whole genome sequencing did not reveal major genomic aberrations it cannot be completely excluded that genetic background differences between the aP2-*ALOX15* mice and wildtype control animals may have contributed to the observed anti-inflammatory effect in the CFA model.

Despite their structural similarities (mouse and human ALOX15 orthologs share a high degree of amino acid conservation) the two ALOX15 orthologs exhibit remarkable functional differences. The human enzyme converts free AA, DHA and ALA mainly to the n-6 hydroperoxy derivatives [63–65]. In contrast, the major oxygenation products of these substrates by the mouse ortholog are the n-9 derivatives [28, 49]. On the other hand, when free LA was offered as substrate both enzymes form a similar product mixture [64]. The molecular basis for the different reaction specificities of the two ALOX15 orthologs has been explored [66–69] and the Triad Concept [2, 70] explains this difference. According to the Evolutionary Hypothesis of mammalian ALOX15 orthologs the vast majority (< 95%) of terrestrial mammals including those of marine mammals [71] express AA 12-lipoxygenating (n-9) ALOX15 orthologs [72]. Interestingly, most mammals ranked in evolution above gibbons including extinct and extant human subspecies express AA 15-lipoxygenating ALOX15 orthologs and these data suggested a systematic alteration of the functional enzyme properties during late mammalian evolution [72]. The evolutionary driving force for the targeted alteration in the reaction specificity of mammalian ALOX15 orthologs is still a matter of discussion but a broader substrate specificity of the AA 15-lipoxygenating and their higher biosynthetic capacity for anti-inflammatory SPMs [72, 73] might confer an evolutionary advantage to *Hominidae*.

Before we started to generate the aP2-*ALOX15* transgenic mice tested here we needed to decide whether we should use human or mouse ALOX15 as transgenic enzyme. Since both ALOX15 orthologs exhibit different functional properties (AA 15-lipoxygenation by human ALOX15 *vs.* AA 12-lipoxygenation by mouse Alox15) this question was very relevant for functional studies of these animals. After internal discussions we decided to employ the human ALOX15 as transgene since according to the evolutionary hypothesis this enzyme constitutes an ALOX15 ortholog, the functional properties of which have been optimized during late primate evolution [72, 73]. This evolutionary optimization might be of particular relevance, when the transgenic mice will be tested in animal inflammation models. AA 15-lipoxygenating enzyme orthologs including human ALOX15 exhibit an improved biosynthetic capacity for specialized pro-resolving mediators compared to AA 12-lipoxygenating orthologs [72, 73]. Thus, transgenic expression of the evolutionary less optimized mouse Alox15 (this enzyme is AA 12-lipoxygenating) was likely to exhibit less pronounced anti-inflammatory effects. On the other hand, no AA 15-lipoxygenating ALOX-isoforms are present in mice. In other words, in mice and in most other mammals the AA cascade is functional in the absence of 15-HETE formation. Transgenic expression of human AA 15-lipoxygenating ALOX15 might lead to disturbance of other pathways of the AA cascade and such disturbance might contribute to the observed effects. In other words, transgenic expression of human ALOX15 may induce more pronounced anti-inflammatory effects (advantage) but it also makes the AA cascade of the aP2 mice more complex (disadvantage).

### Inhibition of the Alox5 pathway might contribute to the anti-inflammatory effect of transgene expression in the CFA-induced inflammation model

As indicated in Figs. 2, 3, 4, and 5 transgenic overexpression of human ALOX15 under the control of the aP2 promoter partly protected the aP2-*ALOX15* mice in the CFA model. Such anti-inflammatory effects of ALOX15 expression have been reported before and in some of these reports the formation of anti-inflammatory SPMs has been discussed as mechanistic reason for the observed effects [38]. When we characterized the Alox5 pathway in peritoneal lavage cells and bone marrow cells of aP2-*LOX15* mice we recently observed that expression of the human *ALOX15* transgene reduced the catalytic activity of endogenous Alox5. If a similar repressive effect exists in the inflamed paws, suppression of the Alox5 pathway by transgenic ALOX15 expression would offer an alternative explanation for the observed anti-inflammatory effect of transgenic ALOX15 expression. To test this hypothesis, we quantified the steady state concentrations of endogenous Alox5 mRNA in the inflamed paw tissue. We observed that Alox5 expression is significantly reduced in the inflamed paw tissue of aP2-*ALOX15* mice when compared with wildtype control animals (Fig. 6). Because of its biosynthesizing capacity for pro-inflammatory leukotrienes ALOX5 is considered a classical pro-inflammatory enzyme [10, 74] and the ALOX15 induced repression of Alox5 expression observed here might contribute to the anti-inflammatory activity of transgene expression.

The finding that introduction ALOX15 transgene into the mouse genome down-regulates the expression of the pro-inflammatory Alox5 may not be considered the exclusive reason for the protection of the aP2-ALOX15 mice in the CFA-induced paw edema model. It is still possible that other mechanisms such as an augmented biosynthesis of anti-inflammatory and/or pro-resolving mediators [11–16] may also contribute. Unfortunately, we do not have the required analytical devices for LC–MS/MS based oxylipidome profiling in our lab and thus corresponding experiments could not be carried out in the frame of this study.

### Variable roles of Alox15 in different types of colitis models

Since mammalian ALOX15 orthologs have been implicated in different inflammation models Alox15^−/−^ mice have previously been tested in DSS-induced colitis. When compared with outbred wildtype control animals female Alox15^−/−^ mice developed less intense inflammatory symptoms and the protective effect of Alox15 deficiency has been related to a less pronounced impairment of the intestinal epithelial barrier function [54]. Considering this data, one would expect that transgenic expression of human ALOX15 would induce a more severe inflammatory reaction. Although the aP2-*ALOX15* mice experienced an elevated loss of body weight (Fig. 7A) and although colon shortening was more pronounced (Fig. 7B) the observed differences between the two genotypes did not reach the level of statistical significance. As possible explanation for these unexpected findings the following reasons may be discussed: (i) In general, systemic knockout of a defined gene cannot always be compensated by transgenic overexpression of the same gene since expression regulation of the endogenous gene might be different for the endogenous gene and for the transgene. (ii) In Alox15^−/−^ mice expression of the endogenous Alox15 is blocked in all cells and tissues. In contrast, in aP2-*ALOX15* mice human ALOX15 is only in a small number of cells and tissues and we did not detect significant expression of the transgene in the gut. (iii) In Alox15^−/−^ mice biosynthesis of 12-HETE is impaired since one of the major 12-HETE synthesizing pathways is silenced. Human ALOX15 forms 15-HETE as major AA oxygenation product [65] and thus, the biosynthetic capacity for 12-HETE, which is downregulated in Alox15^−/−^ mice, may not be re-elevated by transgenic expression of human ALOX15.

Transgenic *fat1* mice, which are rich in endogenous n-3 PUFAs, are protected in the DSS-induced colitis model [75] but additional systemic inactivation of the *Alox15* gene counteracted this protective effect [55]. In contrast to wildtype mice, in which systemic inactivation of the *Alox15* gene protected the animals [54] from the development of inflammatory symptoms the same genetic manipulation leads to more intense inflammatory symptoms in fat1 mice [55]. In other words, systemic inactivation of the Alox15 gene has anti-inflammatory consequences in wildtype mice but pro-inflammatory consequences in *fat1* mice. Thus, the patho-physiological consequence of Alox15 deficiency depends on the functional state of PUFA metabolism.

## Conclusions

Transgenic mice expressing human ALOX15 under the control of the activating protein 2 promoter (aP2-ALOX15 mice) are partly protected in the complete Freund’s adjuvant (CFA)-induced hind-paw inflammation model and these data suggest an anti-inflammatory effect of the transgene enzyme. In contrast, the intensity of inflammatory symptoms was hardly altered when aP2-ALOX15 mice were tested in the dextran-sodium sulfate-induced colitis. Here we even observed a trend for more intense inflammatory symptoms in the aP2-ALOX15 mice. These data suggest that human ALOX15 exhibits variable (pro-inflammatory and anti-inflammatory) functions in different animal inflammation models and this conclusion is consistent with previously published results.

## Data Availability

All data generated or analyzed during this study are included in this published article. The original experimental raw data can be obtained from the authors upon request. The genetically modified mice created for this study (aP2-*ALOX15* mice) can also be obtained from the authors for collaborative experiments in other mouse models of human diseases.

## Abbreviations

ALOX15: Arachidonic acid 15-lipoxygenase
ALOX5: Arachidonic acid 5-lipoxygenase
ALOX15B: Arachidonic acid lipoxygenase 15B
DSS: Dextran sulfate sodium
CFA: Complete Freund’s adjuvants
DSS: Dextran sodium sulfate
SPMs: Specialized pro-resolving mediators
aP2: Activating protein 2
AA: Arachidonic acid
15-HETE: 15-Hydroxy-5*Z*,8*Z*,11*Z*,13*E*-eicosatetraenoic acid
12-HETE: 12-Hydroxy-5*Z*,8*Z*,10*E*,14Z-eicosatetraenoic acid
qRT-PCR: Quantitative real-time polymerase chain reaction
HPLC: High performance liquid chromatography
ANOVA: Analysis of variance

## References

[1] Singh NK, Rao GN (2019). Emerging role of 12/15-Lipoxygenase (ALOX15) in human pathologies. Prog Lipid Res.

[2] Ivanov I, Kuhn H, Heydeck D (2015). Structural and functional biology of arachidonic acid 15-lipoxygenase-1 (ALOX15). Gene.

[3] Ackermann JA, Hofheinz K, Zaiss MM, Kronke G (2017). The double-edged role of 12/15-lipoxygenase during inflammation and immunity. Biochim Biophys Acta.

[4] Colakoglu M, Tuncer S, Banerjee S (2018). Emerging cellular functions of the lipid metabolizing enzyme 15-Lipoxygenase-1. Cell Prolif.

[5] Funk CD, Chen X-S, Johnson EN, Zhao L (2002). Lipoxygenase genes and their targeted disruption. Prostaglandins Other Lipid Mediat.

[6] Kuhn H, Humeniuk L, Kozlov N, Roigas S, Adel S, Heydeck D (2018). The evolutionary hypothesis of reaction specificity of mammalian ALOX15 orthologs. Prog Lipid Res.

[7] An JU, Kim SE, Oh DK (2021). Molecular insights into lipoxygenases for biocatalytic synthesis of diverse lipid mediators. Prog Lipid Res.

[8] Chrisnasari R, Hennebelle M, Vincken JP, van Berkel WJH, Ewing TA (2022). Bacterial lipoxygenases: Biochemical characteristics, molecular structure and potential applications. Biotechnol Adv.

[9] Murphy RC, Gijon MA (2007). Biosynthesis and metabolism of leukotrienes. Biochem J.

[10] Haeggstrom JZ, Funk CD (2011). Lipoxygenase and leukotriene pathways: biochemistry, biology, and roles in disease. Chem Rev.

[11] Ryan A, Godson C (2010). Lipoxins: regulators of resolution. Curr Opin Pharmacol.

[12] Yoo S, Lim J, Hwang S (2013). Resolvins: endogenously-generated potent painkilling substances and their therapeutic perspectives. Curr Neuropharmacol.

[13] Spite M, Claria J, Serhan CN (2014). Resolvins, specialized proresolving lipid mediators, and their potential roles in metabolic diseases. Cell Metab.

[14] Serhan CN, Dalli J, Karamnov S, Choi A, Park CK, Xu ZZ (2012). Macrophage proresolving mediator maresin 1 stimulates tissue regeneration and controls pain. FASEB J.

[15] Serhan CN, Dalli J, Colas RA, Winkler JW, Chiang N (2015). Protectins and maresins: new pro-resolving families of mediators in acute inflammation and resolution bioactive metabolome. Biochim Biophys Acta.

[16] Serhan CN, Levy BD (2018). Resolvins in inflammation: emergence of the pro-resolving superfamily of mediators. J Clin Investig.

[17] Schebb NH, Kuhn H, Kahnt AS, Rund KM, O’Donnell VB, Flamand N (2022). Formation, signaling and occurrence of specialized pro-resolving lipid mediators-what is the evidence so far?. Front Pharmacol.

[18] Kahnt AS, Schebb NH, Steinhilber D (2023). Formation of lipoxins and resolvins in human leukocytes. Prostaglandins Other Lipid Mediat.

[19] Panigrahy D, Gilligan MM, Serhan CN, Kashfi K (2021). Resolution of inflammation: an organizing principle in biology and medicine. Pharmacol Ther.

[20] Dyall SC, Balas L, Bazan NG, Brenna JT, Chiang N, Souza FD (2022). Polyunsaturated fatty acids and fatty acid-derived lipid mediators: Recent advances in the understanding of their biosynthesis, structures, and functions. Prog Lipid Res.

[21] Kuhn H, Belkner J, Wiesner R, Brash AR (1990). Oxygenation of biological membranes by the pure reticulocyte lipoxygenase. J Biol Chem.

[22] Takahashi Y, Glasgow WC, Suzuki H, Taketani Y, Yamamoto S, Anton M (1993). Investigation of the oxygenation of phospholipids by the porcine leukocyte and human platelet arachidonate 12-lipoxygenases. Eur J Biochem.

[23] Rapoport SM, Schewe T (1986). The maturational breakdown of mitochondria in reticulocytes. Biochim Biophys Acta.

[24] van Leyen K, Duvoisin RM, Engelhardt H, Wiedmann M (1998). A function for lipoxygenase in programmed organelle degradation. Nature.

[25] Rademacher M, Kuhn H, Borchert A (2020). Systemic deficiency of mouse arachidonate 15-lipoxygenase induces defective erythropoiesis and transgenic expression of the human enzyme rescues this phenotype. FASEB J.

[26] Wittwer J, Hersberger M (2007). The two faces of the 15-lipoxygenase in atherosclerosis. Prostaglandins Leukot Essent Fatty Acids.

[27] Schneider C, Pozzi A (2011). Cyclooxygenases and lipoxygenases in cancer. Cancer Metastasis Rev.

[28] Sun D, Funk CD (1996). Disruption of 12/15-lipoxygenase expression in peritoneal macrophages. Enhanced utilization of the 5-lipoxygenase pathway and diminished oxidation of low density lipoprotein. J Biol Chem.

[29] Cyrus T, Witztum JL, Rader DJ, Tangirala R, Fazio S, Linton MF (1999). Disruption of the 12/15-lipoxygenase gene diminishes atherosclerosis in apo E-deficient mice. J Clin Invest.

[30] Kronke G, Katzenbeisser J, Uderhardt S, Zaiss MM, Scholtysek C, Schabbauer G (2009). 12/15-lipoxygenase counteracts inflammation and tissue damage in arthritis. J Immunol.

[31] Bleich D, Chen S, Zipser B, Sun D, Funk CD, Nadler JL (1999). Resistance to type 1 diabetes induction in 12-lipoxygenase knockout mice. J Clin Invest.

[32] Reilly KB, Srinivasan S, Hatley ME, Patricia MK, Lannigan J, Bolick DT (2004). 12/15-Lipoxygenase activity mediates inflammatory monocyte/endothelial interactions and atherosclerosis in vivo. J Biol Chem.

[33] Kayama Y, Minamino T, Toko H, Sakamoto M, Shimizu I, Takahashi H (2009). Cardiac 12/15 lipoxygenase-induced inflammation is involved in heart failure. J Exp Med.

[34] Suzuki H, Kayama Y, Sakamoto M, Iuchi H, Shimizu I, Yoshino T (2015). Arachidonate 12/15-lipoxygenase-induced inflammation and oxidative stress are involved in the development of diabetic cardiomyopathy. Diabetes.

[35] Harats D, Shaish A, George J, Mulkins M, Kurihara H, Levkovitz H (2000). Overexpression of 15-lipoxygenase in vascular endothelium accelerates early atherosclerosis in LDL receptor-deficient mice. Arterioscler Thromb Vasc Biol.

[36] Harats D, Ben-Shushan D, Cohen H, Gonen A, Barshack I, Goldberg I (2005). Inhibition of carcinogenesis in transgenic mouse models over-expressing 15-lipoxygenase in the vascular wall under the control of murine preproendothelin-1 promoter. Cancer Lett.

[37] Horvai A, Palinski W, Wu H, Moulton KS, Kalla K, Glass CK (1995). Scavenger receptor A gene regulatory elements target gene expression to macrophages and to foam cells of atherosclerotic lesions. Proc Natl Acad Sci USA.

[38] Merched AJ, Ko K, Gotlinger KH, Serhan CN, Chan L (2008). Atherosclerosis: evidence for impairment of resolution of vascular inflammation governed by specific lipid mediators. FASEB J.

[39] Madison BB, Dunbar L, Qiao XT, Braunstein K, Braunstein E, Gumucio DL (2002). Cis elements of the villin gene control expression in restricted domains of the vertical (crypt) and horizontal (duodenum, cecum) axes of the intestine. J Biol Chem.

[40] Tian R, Zuo XS, Jaoude J, Mao F, Colby J, Shureiqi I (2017). ALOX15 as a suppressor of inflammation and cancer: Lost in the link. Prostaglandins Other Lipid Mediat.

[41] Coppey L, Obrosov A, Shevalye H, Davidson E, Paradee W, Yorek MA (2021). Characterization of mice ubiquitously overexpressing human 15-lipoxygenase-1: effect of diabetes on peripheral neuropathy and treatment with Menhaden oil. J Diabetes Res.

[42] Shen J, Kuhn H, Petho-Schramm A, Chan L (1995). Transgenic rabbits with the integrated human 15-lipoxygenase gene driven by a lysozyme promoter: macrophage-specific expression and variable positional specificity of the transgenic enzyme. FASEB J.

[43] Shen J, Herderick E, Cornhill JF, Zsigmond E, Kim HS, Kuhn H (1996). Macrophage-mediated 15-lipoxygenase expression protects against atherosclerosis development. J Clin Invest.

[44] Song YS, Lee DH, Yu JH, Oh DK, Hong JT, Yoon DY (2016). Promotion of adipogenesis by 15-(S)-hydroxyeicosatetraenoic acid. Prostaglandins Other Lipid Mediat.

[45] Cole BK, Morris MA, Grzesik WJ, Leone KA, Nadler JL (2012). Adipose tissue-specific deletion of 12/15-lipoxygenase protects mice from the consequences of a high-fat diet. Mediators Inflamm.

[46] Lieb DC, Brotman JJ, Hatcher MA, Aye MS, Cole BK, Haynes BA (2014). Adipose tissue 12/15 lipoxygenase pathway in human obesity and diabetes. J Clin Endocrinol Metab.

[47] Heydeck D, Ufer C, Kakularam KR, Rothe M, Liehr T, Poulain P (2023). Functional Characterization of Transgenic Mice Overexpressing Human 15-Lipoxygenase-1 (ALOX15) under the Control of the aP2 Promoter. Int J Mol Sci.

[48] Marbach-Breitruck E, Rohwer N, Infante-Duarte C, Romero-Suarez S, Labuz D, Machelska H (2021). Knock-In mice expressing a 15-lipoxygenating Alox5 mutant respond differently to experimental inflammation than reported Alox5(-/-) Mice. Metabolites.

[49] Freire-Moar J, Alavi-Nassab A, Ng M, Mulkins M, Sigal E (1995). Cloning and characterization of a murine macrophage lipoxygenase. Biochim Biophys Acta.

[50] Brack A, Labuz D, Schiltz A, Rittner HL, Machelska H, Schafer M (2004). Tissue monocytes/macrophages in inflammation: hyperalgesia versus opioid-mediated peripheral antinociception. Anesthesiology.

[51] Chillingworth NL, Donaldson LF (2003). Characterisation of a Freund's complete adjuvant-induced model of chronic arthritis in mice. J Neurosci Methods.

[52] Eichele DD, Kharbanda KK (2017). Dextran sodium sulfate colitis murine model: an indispensable tool for advancing our understanding of inflammatory bowel diseases pathogenesis. World J Gastroenterol.

[53] Perse M, Cerar A (2012). Dextran sodium sulphate colitis mouse model: traps and tricks. J Biomed Biotechnol.

[54] Kroschwald S, Chiu CY, Heydeck D, Rohwer N, Gehring T, Seifert U (2018). Female mice carrying a defective Alox15 gene are protected from experimental colitis via sustained maintenance of the intestinal epithelial barrier function. Biochim Biophys Acta Mol Cell Biol Lipids.

[55] Rohwer N, Chiu CY, Huang D, Smyl C, Rothe M, Rund KM (2021). Omega-3 fatty acids protect from colitis via an Alox15-derived eicosanoid. FASEB J.

[56] Kuhn H, O'Donnell VB (2006). Inflammation and immune regulation by 12/15-lipoxygenases. Prog Lipid Res.

[57] Cathcart MC, Lysaght J, Pidgeon GP (2011). Eicosanoid signalling pathways in the development and progression of colorectal cancer: novel approaches for prevention/intervention. Cancer Metastasis Rev.

[58] Pidgeon GP, Lysaght J, Krishnamoorthy S, Reynolds JV, O'Byrne K, Nie D (2007). Lipoxygenase metabolism: roles in tumor progression and survival. Cancer Metastasis Rev.

[59] Kawai T, Autieri MV, Scalia R (2021). Adipose tissue inflammation and metabolic dysfunction in obesity. Am J Physiol Cell Physiol.

[60] van Leyen K (2013). Lipoxygenase: an emerging target for stroke therapy. CNS Neurol Disord Drug Targets.

[61] Biringer RG (2019). The role of eicosanoids in Alzheimer's disease. Int J Environ Res Public Health.

[62] Zuo X, Peng Z, Wu Y, Moussalli MJ, Yang XL, Wang Y (2012). Effects of gut-targeted 15-LOX-1 transgene expression on colonic tumorigenesis in mice. J Natl Cancer Inst.

[63] Sigal E, Grunberger D, Cashman JR, Craik CS, Caughey GH, Nadel JA (1988). Arachidonate 15-lipoxygenase from human eosinophil-enriched leukocytes: partial purification and properties. Biochem Biophys Res Commun.

[64] Kutzner L, Goloshchapova K, Heydeck D, Stehling S, Kuhn H, Schebb NH (2017). Mammalian ALOX15 orthologs exhibit pronounced dual positional specificity with docosahexaenoic acid. Biochim Biophys Acta.

[65] Kühn H, Barnett J, Grunberger D, Baecker P, Chow J, Nguyen B (1993). Overexpression, purification and characterization of human recombinant 15-lipoxygenase. Biochim Biophys Acta.

[66] Sloane DL, Leung R, Barnett J, Craik CS, Sigal E (1995). Conversion of human 15-lipoxygenase to an efficient 12-lipoxygenase: the side-chain geometry of amino acids 417 and 418 determine positional specificity. Protein Eng.

[67] Sloane DL, Leung R, Craik CS, Sigal E (1991). A primary determinant for lipoxygenase positional specificity. Nature.

[68] Borngraber S, Kuban RJ, Anton M, Kuhn H (1996). Phenylalanine 353 is a primary determinant for the positional specificity of mammalian 15-lipoxygenases. J Mol Biol.

[69] Borngraber S, Browner M, Gillmor S, Gerth C, Anton M, Fletterick R (1999). Shape and specificity in mammalian 15-lipoxygenase active site. The functional interplay of sequence determinants for the reaction specificity. J Biol Chem.

[70] Vogel R, Jansen C, Roffeis J, Reddanna P, Forsell P, Claesson H-E (2010). Applicability of the triad concept for the positional specificity of mammalian lipoxygenases. J Biol Chem.

[71] Reisch F, Kakularam KR, Stehling S, Heydeck D, Kuhn H (2021). Eicosanoid biosynthesis in marine mammals. FEBS J.

[72] Heydeck D, Reisch F, Schäfer M, Kakularam KR, Roigas SA, Stehling S (2022). The reaction specificity of mammalian ALOX15 Orthologs is changed during late primate evolution and these alterations might offer evolutionary advantages for hominidae. Front Cell Dev Biol.

[73] Adel S, Karst F, Gonzalez-Lafont A, Pekarova M, Saura P, Masgrau L (2016). Evolutionary alteration of ALOX15 specificity optimizes the biosynthesis of antiinflammatory and proresolving lipoxins. Proc Natl Acad Sci USA.

[74] Radmark O, Werz O, Steinhilber D, Samuelsson B (2015). 5-Lipoxygenase, a key enzyme for leukotriene biosynthesis in health and disease. Biochim Biophys Acta.

[75] Hudert CA, Weylandt KH, Lu Y, Wang J, Hong S, Dignass A (2006). Transgenic mice rich in endogenous omega-3 fatty acids are protected from colitis. Proc Natl Acad Sci USA.

